# Mutation in *WDR4* impairs tRNA m^7^G_46_ methylation and causes a distinct form of microcephalic primordial dwarfism

**DOI:** 10.1186/s13059-015-0779-x

**Published:** 2015-09-28

**Authors:** Ranad Shaheen, Ghada M H Abdel-Salam, Michael P. Guy, Rana Alomar, Mohamed S. Abdel-Hamid, Hanan H. Afifi, Samira I. Ismail, Bayoumi A. Emam, Eric M. Phizicky, Fowzan S. Alkuraya

**Affiliations:** Department of Genetics, King Faisal Specialist Hospital and Research Center, Riyadh, Saudi Arabia; Clinical Genetics Department, Human Genetics and Genome Research Division, National Research Centre, Cairo, Egypt; Department of Biochemistry and Biophysics, Center for RNA Biology, University of Rochester School of Medicine and Dentistry, Rochester, NY USA; Medical Molecular Genetics Department, Human Genetics and Genome Research Division, National Research Centre, Cairo, Egypt; Department of Anatomy and Cell Biology, College of Medicine, Alfaisal University, Riyadh, Saudi Arabia; Current address: Department of Chemistry, Northern Kentucky University, Highland Heights, KY USA

## Abstract

**Background:**

Primordial dwarfism is a state of extreme prenatal and postnatal growth deficiency, and is characterized by marked clinical and genetic heterogeneity.

**Results:**

Two presumably unrelated consanguineous families presented with an apparently novel form of primordial dwarfism in which severe growth deficiency is accompanied by distinct facial dysmorphism, brain malformation (microcephaly, agenesis of corpus callosum, and simplified gyration), and severe encephalopathy with seizures. Combined autozygome/exome analysis revealed a novel missense mutation in *WDR4* as the likely causal variant. WDR4 is the human ortholog of the yeast Trm82, an essential component of the Trm8/Trm82 holoenzyme that effects a highly conserved and specific (m^7^G_46_) methylation of tRNA. The human mutation and the corresponding yeast mutation result in a significant reduction of m^7^G_46_ methylation of specific tRNA species, which provides a potential mechanism for primordial dwarfism associated with this lesion, since reduced m^7^G_46_ modification causes a growth deficiency phenotype in yeast.

**Conclusion:**

Our study expands the number of biological pathways underlying primordial dwarfism and adds to a growing list of human diseases linked to abnormal tRNA modification.

**Electronic supplementary material:**

The online version of this article (doi:10.1186/s13059-015-0779-x) contains supplementary material, which is available to authorized users.

## Background

Primordial dwarfism (PD) is a term used to describe a wide range of phenotypes that have in common severe prenatal growth deficiency (>3 SD below the mean) that persists postnatally [1]. Although extremely rare, the monogenic nature of PD lends itself readily to gene mapping approaches thus representing a unique resource for understanding the biological networks that control growth through the discovery of genes that are mutated in this condition [2].

Impaired DNA damage repair is among the earliest identified mechanisms in PD as revealed by the discovery that *ATR* is mutated in patients with Seckel syndrome, a clinical subtype of PD characterized by microcephaly and distinct facial features [3]. The same mechanism is invoked in PD caused by mutations in *ATRIP*, *BRCA2*, *DNA2*, and *XRCC4* [4, 5]. Impaired mitosis due to centrosomal abnormalities has now emerged as a major mechanism underlying many forms of PD [2, 6–9]. Less common forms of PD were found to be caused by mutations in genes involved in replication licensing, splicing and serine synthesis [10–13]. Despite the remarkable acceleration of PD disease gene discovery in recent years, one-third of the cases remain undiagnosed molecularly, which suggests that additional disease genes likely exist and these might further expand the known molecular network that controls growth [4].

tRNA is a well-studied class of non-coding RNA that plays an essential role in protein synthesis by transferring amino acids to the growing peptide chain as the corresponding mRNA is being decoded by the ribosomal translational machinery. A remarkable multitude of modification reactions (>100) are known, which are often highly conserved in different organisms, including in prokaryotes and archaea, clearly suggesting their importance [14]. Our knowledge of the biology of tRNA modification comes primarily from work on the yeast *Saccharomyces cerevisiae* and other model organisms [15–17]. In general, modifications in the tRNA anticodon loop are critical for translational efficiency, frame maintenance, and fidelity, and lack of these modifications often leads to lethality, slow growth, and/or other phenotypic effects [16, 18]. Modifications to the body of the tRNA are generally involved in tRNA folding and stability [19–22], and lack of any of several different body modifications in yeast causes temperature sensitivity due to rapid tRNA decay (RTD) of specific tRNAs [23–25].

The recent identification of several links between tRNA modification and human disease have spurred increased interest in this field and its potential to explain the pathogenesis of clinically relevant disorders [26]. In this study, we describe an apparently novel clinical condition characterized by primordial dwarfism and a unique set of additional features. We show that the two families affected by this disorder map to *WDR4*, the human ortholog of Trm82, which is required for formation of the highly conserved m^7^G_46_ (7-methylguanosine) modification of tRNA. The m^7^G_46_ modification occurs widely in prokaryotes and eukaryotes [14], and in *S. cerevisiae* requires a holoenzyme comprised of the Trm8 methyltransferase subunit and its WD40 repeat-containing binding partner Trm82 [27], which appears to be involved in maintaining Trm8 levels [28], and in helping Trm8 maintain an active conformation [29]. Yeast *trm82Δ* mutants, like *trm8Δ* mutants, are mildly temperature sensitive due to lack of m^7^G in their tRNA [28], and have synthetic genetic interactions with *trm4Δ* mutants (lacking m^5^C), and a number of other modification mutants, resulting in a severe temperature sensitive growth defect [23]. Human *WDR4* and *METTL1* are the likely orthologs of *S. cerevisiae TRM82* and *TRM8* based on homology, and on their complementation of yeast mutants lacking m7G46 [27]. Here we show that the *WDR4* mutation affects m^7^G_46_ methylation suggesting a potential mechanism for this novel form of PD.

## Results

### Identification of a novel PD syndrome

#### Patient 1 (14DG1157)

**Fig. 1:**
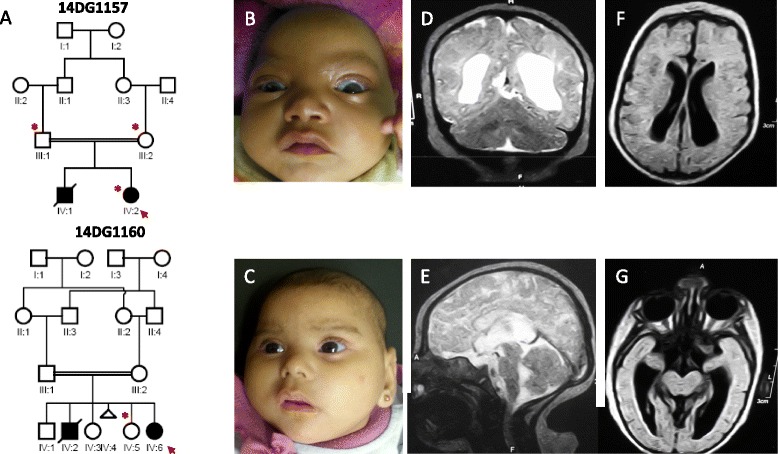
Identification of a novel PD syndrome. **a** Family pedigree of 14DG1157 & 14DG1160 showing the consanguineous nature of the parents. The index is indicated in each pedigree by an arrow, and asterisks denote individuals whose DNA was available for analysis. **b, c** Facial images for the index of each family showing the highly similar dysmorphic profile consisting of high forehead, prominent eyes, depressed nasal bridge, short philtrum, tented upper lip and bulged alveolar ridge, and prominent ear lobule. **d**–**g** MRI image of 14DG1160 showing partial agenesis of corpus callosum, and abnormal gyral pattern most pronounced posteriorly

This female infant was born to a 20-year-old mother and 26-year-old father by normal vaginal delivery at 37-weeks’ gestation. The parents are healthy first cousins (Fig. 1a). The family history is non-contributory. The couple had a subsequent boy who died a few days after birth because of growth retardation and multiple congenital heart anomalies. During the gestation of patient 1, the pregnancy was complicated by threatened abortion in the first trimester. Intrauterine growth retardation and weak fetal movements were also documented. The birth weight was 1,600 g. The birth length and head circumference were not recorded but mentioned to be small. She was referred to the Clinical Genetics Department at the age of 4 months because of poor gain of weight and for genetic counseling. On clinical examination she was noted to have a head circumference of 31.5 cm (−5 SD) weight of 2,800 g (−6 SD), and length of 48 cm (−5 SD). The patient had a high forehead, prominent eyes, depressed nasal bridge, short philtrum, tented upper lip and bulged alveolar ridge, and prominent ear lobule (Fig. 1b).

Her neurological evaluation revealed hypertonia, brisk deep-tendon reflexes, with flexor plantar responses. At that age, radiological examination showed unossified pubic bones, proportionately short femora, and broad metaphyses of the femora and tibiae. The tibiae and fibulae were short and equal in length.

At the age of 10 months, she developed seizures with only partial response to valproate and lamotrigine. EEG records revealed low voltage slow waves (3–6 cycles/s) mixed with sleep spindle.

At the age of 20 months, her weight, length, and head circumference were 3,500 g (−6.5 SD), 55 cm (−9.5 SD), and 32 cm (−10.7 SD), respectively. The girl was spastic with contracture deformity in the elbows and hands. Her neurological evaluation revealed hypertonia, brisk deep-tendon reflexes, with flexor plantar responses. She had not acquired any developmental milestones and never recognized her mother. Abdominal ultrasonography and echocardiography showed normal results. Ophthalmological examination showed bilateral optic atrophy.

Chromosomal examination from peripheral blood lymphocytes and high resolution banding technique revealed normal female karyotype 46,XX.

Cranial MRI showed partial agenesis of corpus callosum, and abnormal gyral pattern most pronounced posteriorly.

Diagnosis of microcephalic primordial dwarfism was made at this time based on significant pre- and post-natal growth retardation. Because of the partial overlap with microcephalic osteodysplastic primordial dwarfism I, analysis of *RNU4ATAC* was undertaken but revealed negative results.

#### Patient 2 (14DG1160)

Patient 2 was the fifth child born to a 36-year-old mother and 39-year-old father. The parents are maternal and paternal first cousins (double consanguineous) (Fig. 1a). The second pregnancy resulted in a similarly affected boy who died at the age of 9 months because of pneumonia accompanied by uncontrolled seizures. A postmortem examination was not performed. There was no other family history of note. Patient 2 was delivered vaginally weighing 1,500 g (−3.7 SD) at 38 weeks’ gestation. Her birth length and head circumference were not recorded. At age 4 months she experienced focal seizures that later evolved into generalized tonic-clonic seizures controlled by combination sodium valproate, levetiracetam, and lamotrigine therapy. EEG records revealed high voltage delta waves (2–4 cycles/s). Abdominal ultrasonography and echocardiography showed normal results.

At the age of 7 months, she was referred to our Clinical Genetics Department because of the microcephaly and poor weight gain for genetic counseling. On examination, her weight, length and head circumference were 4,200 g (−4.7 SD), 53 cm (−5.7 SD), and 33.2 cm (−7 SD), respectively. Her facial features were quite similar to patient 1 showing rounded face with high forehead, prominent eyes, depressed nasal bridge, short philtrum, bow shaped mouth, and prominent alveolar ridge (Fig. 1c). Her neurological evaluation revealed hypertonia, brisk deep-tendon reflexes, with flexor plantar responses. Ophthalmological examination showed bilateral optic atrophy.

She presented at the age of 9 months with high fever and chest infection that diagnosed as pneumonia. This was accompanied by status epilepticus and she went into coma for 30 days.

Follow-up at the age of 17 months, her weight, length, and head circumference were 6,500 g (−3.8 SD), 60 cm (−6.7 SD), and 34.5 cm (−8.9 SD), respectively. The girl made almost no developmental progress and could not recognize the surroundings. Oro-dental examination showed thick alveolar ridge more in the upper than lower and high arched palate.

Routine biochemical and metabolic screening parameters were within normal ranges.

Chromosomal examination from peripheral blood lymphocytes and high resolution banding technique revealed normal female karyotype 46,XX. Radiographic examination of the long bones showed proportionately short long bones with broad metaphyses.

Cranial MRI showed partial agenesis of corpus callosum, and abnormal gyral pattern most pronounced posteriorly (Fig. 1d–g).

No developmental progress was observed on the last examination of both patients. They were not able to raise their heads or roll, and never laughed. They were unable to follow visually, recognize their mothers, or make eye-to-eye contact. There were no vocalizations beyond an infrequent moaning when in discomfort. Patient 2 sometimes required a tube feeding at the age of 9 months.

### A novel PD syndrome maps to a founder mutation in *WDR4*

**Fig. 2:**
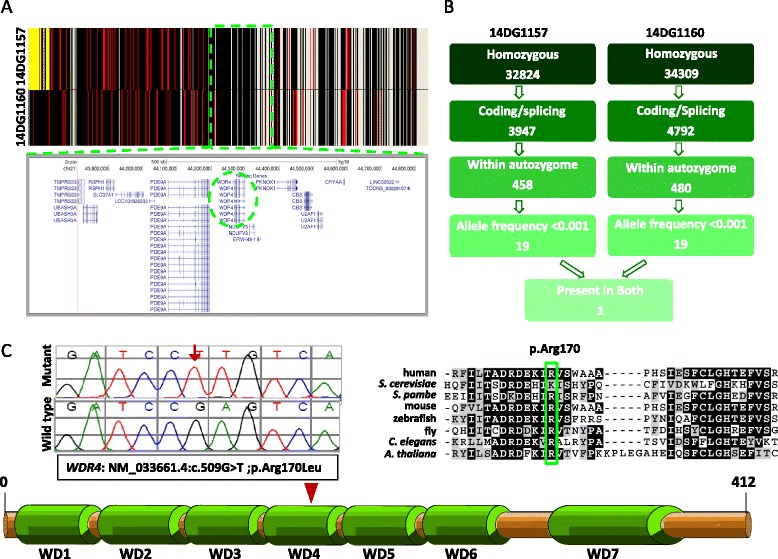
A novel PD syndrome is linked to a founder mutation in WDR4. **a** AutoSNPa showing the shared haplotype between individual II:2 in Family 1, Individual II:6 in Family 2 denoted by black lines (boxed in green lines). **b** illustration to the exome filtering scheme and the number of survived variants in each step in both **c** Sequence chromatograms of the mutation (control tracing is shown for comparison and the location of the mutation is denoted by an arrow) and its location indicated in WD repeat domain 4 on a schematic of WDR4. Also shown is the multisequence alignment of the mutated reside (p.Arg170) showing high conservation down to *Danio rerio* (boxed in green)

Although the two families have different surnames, they come from the same geographic location in Egypt, raising the possibility of a founder mutation. Indeed, autozygosity mapping and haplotype analysis revealed a single shared homozygous haplotype between the two available patients (chr21:43,809,418-44,828,031 (GRCh37/hg19)) spanning 14 RefSeq genes (Fig. 2a). Whole exome sequencing (WES) was performed separately on each index and the resulting variants were filtered based on frequency (novel or <0.0001), zyogsity (homozygous), position (within the autozygome of the corresponding samples), and nature of the variant (coding/splicing, excluding synonymous changes and those predicted to be benign by two independent *in silico* prediction tools). Although each index had a few variants that survived these filters, only one variant was shared by the two (*WDR4*, NM_033661.4:c.509G > T; p.Arg170Leu) (Fig. 2b, c, Additional file 1: Table S1). Reassuringly, this variant was also within the single shared homozygous haplotype, that is, this is the only novel coding/splicing homozygous variant within the critical locus (Additional file 2: Table S2). Segregation analysis using available family members confirmed that only the two patients were homozygous. This mutation is absent in 615 in-house Saudi exomes, 1000 Genomes, Exome Server, and ExAC Browser. It is predicted to be pathogenic by PolyPhen (0.993), SIFT (0), and CADD (PHRED: 20.3).

### Yeast *trm82-K223L* mutants have decreased levels of m^7^G_46_ on tRNA

**Fig. 3:**
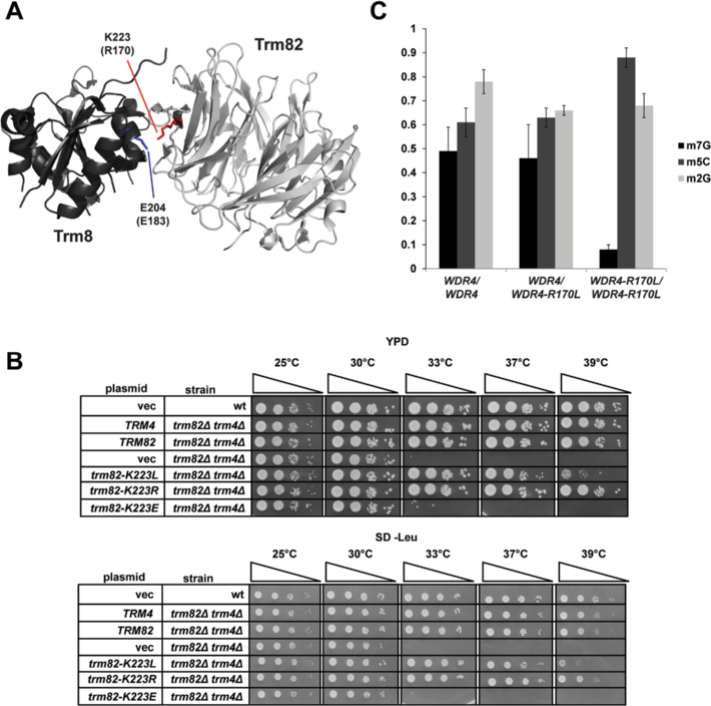
The *WDR4-R170L* mutation results in decreased levels of m^7^G on tRNA. **a** Predicted location of R170 residue of WDR4, based on the yeast Trm8-Trm82 crystal structure. Representation of *S. cerevisiae* Trm8-Trm82 holoenzyme (PDB 2VDU), Trm82 is light gray with residue K223 in red, and Trm8 is in dark gray with residue E204 in blue. Corresponding WDR4 and METTL1 residues are in brackets. **b** A *trm82*Δ *trm4*Δ (*CEN LEU2* P_*TRM82*_ *-trm82-K223L*) strain has a growth defect at high temperature. Wild type and *trm82*Δ *trm4*Δ strains with (*LEU2*) plasmids expressing *TRM82* variants as indicated were grown overnight in S -Leu medium containing dextrose, diluted to OD_600_ of approximately 0.5 in H_2_O, and serially diluted 10-fold in H_2_O, and then 2 μL was spotted onto indicated media, followed by incubation for 3 days as indicated. **c** Nucleoside analysis of tRNA^Phe^ purified from human LCLs derived from a PD patient homozygous for the *WDR4-R170L* allele. tRNA^Phe^ isolated from LCLs derived from the *WDR4-R170L* proband with PD, and from control LCLs, was digested to nucleosides and analyzed by HPLC as described in Materials and Methods

We reasoned that homozygosity of the *WDR4-R170L* allele would result in defective m^7^G_46_ modification of tRNA, since *WDR4* is the likely human ortholog of *S. cerevisiae* Trm82 [27], since the corresponding residue in other eukaryotes is almost always an arginine and occasionally a lysine, since this residue is within the most highly conserved region of the Trm8/WDR4 family (Fig. 2c), and since residue K223 of yeast Trm82 (corresponding to R170 in human WDR4) forms a salt bridge with residue E204 of Trm8 that is speculated to be important for maintaining Trm8 in an active conformation (Fig. 3a) [29]. We therefore used *S. cerevisiae* as a model to analyze the effects of the *WDR4-R170L* mutation. We generated a low copy (*CEN*) plasmid expressing the *S. cerevisiae trm82-K223L* from its native promoter (*CEN LEU2* P_*TRM82*_-*trm82-K223L*) to test its ability to complement an *S. cerevisiae trm82Δ* mutant in a *trm4Δ* background, to amplify the growth defects of the *trm82Δ* mutation [23, 28]. We also generated plasmids bearing a *trm82-K223R* variant to determine if, as expected, arginine and lysine residues are interchangeable at this location, and a *trm82-K223E* variant to test the effects of completely abrogating the salt bridge.

We found that expression of *trm82-K223L* suppressed the growth defect of the *trm82Δ trm4Δ* strain at temperatures up to 37 °C on rich (YP + dextrose) or minimal medium (S -Leu + dextrose), but not at 39 °C (Fig. 3b), indicating that the Trm82-K223L variant is defective for function at high temperature, but is not a complete loss-of-function mutation. By contrast, expression of *trm82-K223E* did not suppress the growth defect of a *trm82Δ trm4Δ* strain at any temperature, consistent with a null phenotype, whereas expression of *trm82-K223R* suppressed the defect at all temperatures (Fig. 3b), demonstrating that arginine and lysine are interchangeable at this residue.

To determine the extent to which m^7^G levels were affected in the *trm82-K223L* mutant (*trm82Δ* (*CEN LEU2* P_*TRM82*_-*trm82-K223L*)), we analyzed modification levels of two of the 11 yeast tRNA species known to have m^7^G after growth at 30 °C or 37 °C, by purification of the corresponding tRNA species from bulk RNA, followed by nuclease digestion and analysis of nucleosides by HPLC. Strikingly, we found that tRNA^Phe^ from the *trm82-K223L* mutant had no detectable m^7^G after growth at 37 °C, but only mildly reduced m^7^G levels after growth at 30 °C, compared to levels in the wild type or the *trm82-K223R* controls (0.21 moles/mole vs. 0.34 or 0.32, respectively; Table 1). Other modifications of tRNA^Phe^ were unaffected in the *trm82* mutants. By contrast, we observed a milder reduction in m^7^G levels of tRNA^Val(AAC)^ from the *trm82-K223L* strain after growth at 37 °C, compared to that from the wild type or *trm82-K223R* controls (0.25 moles/mole vs. 0.50 and 0.55, respectively; Table 1), and no obvious reduction in m^7^G levels of tRNA^Val(AAC)^ when strains were grown at 30 °C (0.45 moles/mole vs. 0.53 and 0.46, respectively; Table 2). The temperature-sensitive reduction in m^7^G levels found on both tRNA^Phe^ and tRNA^Val(AAC)^ from the *trm82-K223L* mutant is consistent with its temperature sensitive complementation of a *trm82Δ trm4Δ* strain (Fig. 3b).

**Table 1:** HPLC analysis of tRNA^Phe^ nucleoside content from an *S. cerevisiae trm82∆* strain expressing *TRM82* variants

Mod.	Mol. exp.	wt (*CEN* vec)	*trm82∆* (*CEN TRM82*)	*trm82∆* (*CEN* vec)	*trm82∆* (*CEN trm82-K223L*)	*trm82∆* (*CEN trm82-K223R*)
30 °C						
m^7^G	1	0.41 ± 0.04	0.34 ± 0.03	<0.03	0.21 ± 0.01	0.32 ± 0.04
Ψ	2	2.09 ± 0.06	1.94 ± 0.03	1.85 ± 0.04	1.99 ± 0.07	2.06 ± 0.05
Cm	1	0.89 ± 0.09	0.92 ± 0.05	0.93 ± 0.08	0.95 ± 0.03	0.93 ± 0.06
Gm	1	0.72 ± 0.01	0.76 ± 0.07	0.76 ± 0.03	0.73 ± 0.08	0.75 ± 0.02
m^5^C	2	1.53 ± 0.06	1.64 ± 0.07	1.69 ± 0.03	1.66 ± 0.06	1.59 ± 0.03
m^2^G	1	0.78 ± 0.02	0.83 ± 0.04	0.87 ± 0.02	0.83 ± 0.04	0.82 ± 0.03
37 °C						
m^7^G	1	0.37 ± 0.03	0.21 ± 0.03	<0.03	<0.03	0.18 ± 0.02
Ψ	2	2.00 ± 0.08	1.91 ± 0.06	1.87 ± 0.01	1.82 ± 0.10	1.92 ± 0.01
Cm	1	0.98 ± 0.08	1.00 ± 0.14	0.91 ± 0.02	0.93 ± 0.05	0.90 ± 0.04
Gm	1	0.77 ± 0.03	0.84 ± 0.02	0.91 ± 0.05	0.96 ± 0.02	0.84 ± 0.02
m^5^C	2	1.63 ± 0.03	1.69 ± 0.02	1.79 ± 0.05	1.76 ± 0.04	1.71 ± 0.02
m^2^G	1	0.82 ± 0.05	0.88 ± 0.02	0.90 ± 0.07	0.94 ± 0.06	0.86 ± 0.02

Mean and standard deviation based on three individual growths and RNA preparations

**Table 2:** HPLC analysis of tRNA^Val(AAC)^ nucleoside content from an *S. cerevisiae trm82∆* strain expressing *TRM82* variants

Mod.	Mol. exp.	wt (*CEN* vec)	*trm82∆* (*CEN TRM82*)	*trm82∆* (*CEN* vec)	*trm82∆* (*CEN trm82-K223L*)	*trm82∆* (*CEN trm82-K223R*)
30 °C						
m^7^G	1	0.50 ± 0.07	0.53 ± 0.03	<0.03	0.45 ± 0.05	0.46 ± 0.07
Ψ	4	3.66 ± 0.10	3.87 ± 0.18	3.94 ± 0.18	3.89 ± 0.15	3.64 ± 0.08
I	1	0.76 ± 0.02	0.85 ± 0.07	0.91 ± 0.06	0.90 ± 0.05	0.77 ± 0.01
m^5^C	1	0.87 ± 0.03	0.94 ± 0.03	1.14 ± 0.05	0.97 ± 0.05	0.91 ± 0.03
m^1^G	1	0.81 ± 0.05	0.98 ± 0.05	0.99 ± 0.05	1.00 ± 0.08	0.83 ± 0.05
m^2^G	0	0.03 ± 0.01	0.05 ± 0.01	0.54 ± 0.10	0.07 ± 0.02	0.05 ± 0.01
37 °C						
m^7^G	1	0.61 ± 0.04	0.50 ± 0.07	<0.03	0.25 ± 0.03	0.55 ± 0.11
Ψ	4	3.88 ± 0.10	3.93 ± 0.05	3.85 ± 0.15	3.99 ± 0.20	4.10 ± 0.08
I	1	0.80 ± 0.01	0.77 ± 0.02	0.74 ± 0.06	0.78 ± 0.07	0.85 ± 0.04
m^5^C	1	1.04 ± 0.04	1.19 ± 0.04	1.53 ± 0.08	1.42 ± 0.09	1.16 ± 0.07
m^1^G	1	0.87 ± 0.01	0.79 ± 0.03	0.79 ± 0.10	0.87 ± 0.06	0.94 ± 0.01
m^2^G	0	0.05 ± 0.03	0.10 ± 0.03	0.45 ± 0.03	0.27 ± 0.05	0.06 ± 0.01

Mean and standard deviation based on three individual growths and RNA preparations

We note that growth of the trm82*Δ* and *trm82-K223L* strains at 37 °C resulted in substantially increased levels of m^5^C on tRNA^Val(AAC)^ (Table 2), similar to the increase in m^5^C levels observed previously when wild type cells were grown under stress conditions [30–32]. Unexpectedly, tRNA^Val(AAC)^ in the *trm82Δ* mutant also had acquired an m^2^G modification (0.54 and 0.45 moles/mole at 30 °C and 37 °C, respectively, compared to barely detectable levels in the wild type strain). Since tRNA^Val(AAC)^ in the *trm82-K223L* strain acquired 0.17 moles/mole m^2^G at 37 °C relative to wild type (from 0.10 to 0.27 moles/mole) while m^7^G levels were reduced by 0.25 moles/mole (from 0.50 to 0.25), we speculate that m^2^G levels inversely correlate with m^7^G levels on this tRNA. Other tRNA^Val(AAC)^ modifications (pseudouridine, inosine, and m^1^G) were unaffected by the *trm82* mutations (Table 2).

### Cells derived from a patient homozygous for the *WDR4-R170L* allele have decreased levels of m^7^G on tRNA

To further define the defect of the *WDR4-R170L* allele and its relationship to PD, we analyzed the m^7^G levels in tRNA^Val(AAC)^ and tRNA^Phe^ purified from lymphoblastoid cell lines (LCLs) derived from the PD patient 1 (14DG1160), as well as from control LCLs derived from the healthy mother (heterozygous) and a healthy brother (homozygous wild type *WDR4*). We found a substantial decrease in m^7^G on tRNA^Phe^ from the PD LCL (0.08 moles/mole in the *WDR4-R170L* homozygote vs. 0.49 and 0.46 for the homozygous wild type *WDR4* LCL and the heterozygous LCL, respectively; Fig. 3c; Table 3), similar to m^7^G levels in tRNA^Phe^ from the corresponding yeast *trm82-K223L* mutant (Table 1). By contrast, levels of the control tRNA^Phe^ modifications Cm, Gm, m^2^G, pseudouridine, and m^5^C modifications were similar in all of the LCLs. We also observed a minor reduction of m^7^G on tRNA^Val(AAC)^ (0.35 moles/mole in the WDR4-R170L homozygote vs. 0.52 and 0.46 for the wild type *WDR4* homozygote and heterozygotes, respectively; Table 3), with similar amounts of each of four control modifications observed in the three different LCLs. Thus, our results strongly suggest that the *WDR4-R170L* mutation causes defects in m^7^G_46_ modification in the PD patients.

**Table 3:** HPLC analysis of tRNA^Phe^ and tRNA^Val(AAC)^ nucleoside content from human LCLs

Mod.	Mol. exp.	*WDR4/WDR4*	*WDR4/wdr4-R170L*	*wdr4-R170L/wdr4-R170L*
tRNA^Phe^				
m^7^G	1	0.49 ± 0.10	0.46 ± 0.14	0.08 ± 0.02
Ψ	4	3.52 ± 0.06	3.82 ± 0.15	3.53 ± 0.15
Cm	1	0.71 ± 0.10	0.73 ± 0.02	0.86 ± 0.03
Gm	1	0.71 ± 0.07	0.63 ± 0.20	0.70 ± 0.17
m^5^C	1	0.61 ± 0.06	0.63 ± 0.04	0.88 ± 0.04
m^2^G	1	0.78 ± 0.05	0.66 ± 0.02	0.68 ± 0.05
tRNA^Val(AAC)^				
m^7^G	1	0.52 ± 0.08	0.46 ± 0.02	0.35 ± 0.06
Ψ	3	3.10 ± 0.08	3.07 ± 0.11	2.94 ± 0.16
I	1	0.40 ± 0.03	0.39 ± 0.01	0.33 ± 0.04
m^5^C	2	1.66 ± 0.09	1.70 ± 0.09	1.58 ± 0.20
m^2^G	1	0.90 ± 0.09	0.87 ± 0.11	0.76 ± 0.01

Mean and standard deviation based on three individual growths and RNA preparations

## Discussion

Our results demonstrate a clear temperature sensitivity in yeast caused by the *trm82-K223L* mutation, as reflected by m^7^G levels in both tRNA^Phe^ and tRNA^Val(AAC)^, and a clear importance of the identity of residue 223, based on the complete lack of complementation of a *trm82-K223E* mutant. These results cause us to speculate that the Trm82-K223 variant might itself be temperature sensitive, or have a temperature sensitive interaction with Trm8, through the salt bridge described between K223 of Trm82 and E204 of Trm8 [29]. It also seems highly likely that the *WDR4-R170L* allele encodes a protein with similar biochemical properties to the Trm82-K223L variant, based on the high degree of conservation between these regions of Trm82 and WDR4, as well as the similarly reduced levels of m^7^G modification in the *WDR4-R170L* LCLs.

Several human disorders have been associated with perturbation of tRNA modification, although the level of evidence in support of causal links varies. For example, several tRNA modification genes have been found to be significantly dysregulated in various cancers and some have even been used as specific biomarkers, for example, TRMT2A in breast cancer [33]. Associations between variants in genes involved in tRNA modifications and some phenotypes have been reported, for example, *ELP4* and epilepsy, and *IKBKAP* and bronchial asthma [34, 35]. A more directly causal connection was established for several Mendelian disorders. We previously reported that a point mutation in *ADAT3* causes intellectual disability [36]. *ADAT3* encodes the likely homolog of yeast *TAD*3, which is the non-catalytic subunit of the complex required for I_34_ modification of substrate tRNAs [37]. Thus, the association of *WDR4* with PD is the second report of a mutation in the non-catalytic subunit of a tRNA modification enzyme that results in disease. More closely related to the PD phenotype we report in this paper are previously reported mutations in *TRM10A*, the likely human homolog of yeast *TRM10* required for m^1^G_9_ modification [38], which cause microcephaly and short stature [39, 40]. Similarly, mutations in human *NSUN2*, which is required for m^5^C modification of body residues 48, 49, and 50 in mammals, as well as C_34_ of the anticodon [41, 42], cause variable phenotypes that include microcephaly as a feature [41, 43].

The pathogenesis of Mendelian diseases caused by tRNA modification genes remains unclear but the predilection to CNS involvement [26, 44] raises interesting possibilities about the vulnerability of the brain to any perturbation in the tight regulation of tRNA modification and, presumably, the consequences of such perturbation on protein synthesis. The mutation we previously reported in *ADAT3*, for example, is now the single most common cause of autosomal recessive intellectual disability in Saudi Arabia [45]. In this regard, the neurological phenotype we observed in patients with *WDR4* mutation (severe microcephaly, agenesis of corpus callosum, and lissencephaly) appears to follow the same pattern of bias towards brain involvement.

Severe growth deficiency in the syndrome we describe is not limited to the brain but rather generalized, resulting in microcephalic primordial dwarfism. It is unclear if this phenotype is caused by reduced proliferation, increased apoptosis or a combination of the two. Our finding that LCLs derived from a PD patient homozygous for the *WDR4-R170L* allele had drastically reduced levels of m^7^G in their tRNA^Phe^ and modestly reduced m^7^G in their tRNA^Val(AAC)^ strongly suggests that defective modification is a major contributor to the disease pathogenesis in these patients. However, it is not clear if the PD is caused specifically by the m^7^G defects in tRNA^Phe^ and/or tRNA^Val(AAC)^, by defects in one or more of the other five human tRNA species known to have m^7^G_46_ [14], or by defects in other uncharacterized tRNA species. We note that yeast mutants lacking modifications often have growth defects due to only a subset of the tRNA species lacking those modifications [46].

The reduction in m^7^G levels in human tRNAs might result in degradation of one or more specific tRNA species, resulting in reduced or aberrant translation. Yeast mutants lacking m^7^G, or m^7^G and m^5^C, in their tRNA are temperature sensitive for growth due to 5'-3' exonucleolytic degradation of tRNA^Val(AAC)^ by the RTD pathway, which degrades the tRNA because of its more exposed 5' end [23–25]. Similarly, HeLa cells treated with siRNA to *METTL1* and *NSUN2* to reduce m^7^G and m^5^C modification undergo loss of tRNA^Val(AAC)^ and an accompanying loss of cell viability upon 5-fluorouracil treatment [47]. Reduced levels of specific tRNA species due to reduced m^7^G levels would be expected to lead to defects in translation and/or reduced growth. However, it is not known if there are other translation effects specifically due to reduced levels of m^7^G on tRNA, as has been reported for some modifications in the anticodon loop under certain growth conditions [48].

## Conclusion

Our demonstration that PD patients with the *WDR4-R170L* missense mutation have defects in m^7^G levels on tRNA adds to the list of nine known or predicted human modification genes for which mutations have been strongly linked to human disease or pathologies [36, 39–41, 43, 49–60]. The underlying molecular causes of these disease associations, and why specific modification defects appear to result in specific disease manifestations remains to be determined. Interestingly, there are forms of microcephalic PD caused by impaired splicing [10], presumably resulting in impaired protein synthesis, and this may also be invoked as a potential mechanism in PD patients with abnormal tRNA modification although future research will be required to investigate that possibility.

## Materials and methods

### Human subjects

PD was defined as growth parameters >3 SD below the mean at birth that persist postnatally. Patients were enrolled only after their parents signed a written informed consent form under an IRB-approved protocol (KFSHRC RAC#2080006). Venous blood was collected in EDTA tubes and, when possible, in sodium heparin tubes for DNA extraction and LCL establishment, respectively. The research was carried out in accordance with the principles of the Declaration of Helsinki.

### Autozygosity mapping and exome sequencing

Autozygosity mapping was performed as described before [61]. Briefly, genomewide genotyping using Axiom SNP chip array was followed by mapping of runs of homozygosity (ROH) >2 Mb in size as surrogates of autozygosity using AutoSNPa [62], which also delineates the haplotype structure thus allowing the detection of haplotype sharing across families. Exome capture was performed using TruSeq Exome Enrichment kit (Illumina) following the manufacturer’s protocol. Samples were prepared as an Illumina sequencing library, and in the second step, the sequencing libraries were enriched for the desired target using the Illumina Exome Enrichment protocol. The captured libraries were sequenced using Illumina HiSeq 2000 Sequencer. The reads were mapped against UCSC hg19 (http://genome.ucsc.edu/) by BWA (http://bio-bwa.sourceforge.net/). The SNPs and Indels were detected by SAMTOOLS (http://samtools.sourceforge.net/). Variants from WES were filtered such that only novel (or very low frequency 0.1 %), coding/splicing, homozygous variants that are within the autozygome of the affected fetus and are predicted to be pathogenic were considered as likely causal variants [63]. Frequency of variants was determined using publically available variant databases (1000 Genomes, Exome Variant Server, and ExAC) as well as a database of 630 in-house ethnically-matched exomes. Pathogenicity was likely if the mutation is loss of function (splicing/truncating) or, in the case of missense/in-frame indels, removes a highly conserved amino acid and is predicted to be pathogenic by the three *in silico* prediction modules PolyPhen, SIFT, and CADD.

### Yeast strains and plasmids

Wild-type BY4741 (*MAT*a *his3Δ1 leu2Δ0 met15Δ0 ura3Δ0*), its *trm82Δ::kanMX* derivative, and the homozygous *trm82Δ::natMX trm4Δ::kanMX* diploid (AA0176) were described previously [23], as was the (*CEN LEU2* P_*TRM82*_*-TRM82*) plasmid (AVA0279). Plasmids expressing the *S. cerevisiae TRM82-K223L* (pMG569A), *TRM82-K223R* (pMG570A), and *TRM82-K223E* (pMG571A) variants were generated by QuickChange PCR according to manufacturer’s instructions (Stratagene), and the variant gene was then ligated into the original parent vector to eliminate mutations in the vector that could be introduced by PCR. All plasmids were confirmed by sequencing before use.

### Isolation and purification of tRNA from human and yeast cells

LCLs were grown at 37 °C in 5 % CO_2_ in RPMI 1640 medium containing FBS (15 %), penicillin (1 U/mL), streptomycin (1 μg/mL), and amphotericin b (0.5 μg/mL) to a density of approximately 1.0 × 10^6^ cells/mL, and bulk RNA from approximately 3.6 × 10^8^ cells was extracted with TRIzol (Life Technologies) according to manufacturer's instructions. *S. cerevisiae* strains were grown at indicated temperatures to mid-log phase in synthetic (S) dropout media containing dextrose, and bulk low molecular weight RNA was extracted from 300 OD-mL pellets as previously described [38]. For purification of individual tRNAs, appropriate 5’ biotinylated oligonucleotides were used to first purify tRNA^Phe^ from RNA preparations as previously described [38], followed by purification of tRNA^Val(AAC)^ from the remaining bulk RNA.

### HPLC analysis of tRNA

Purified tRNA was digested with P1 nuclease and phosphatase as previously described [38], and nucleosides were subjected to HPLC analysis at pH 7.0 as previously described [64].

### Data availability

Data used in this paper come from a small and well-defined family. To protect the identity of individuals, these confidential data are not publicly available.

## Consent

Written informed consent was obtained from the patient’s guardian/parent/next of kin for the publication of this report and any accompanying images.

## Additional files

Additional file 1: Table S1. List of survived rare variants observed in each patient after applying the various filters. (XLSX 12 kb) Additional file 2: Table S2. The SNP calls in the haplotype around the mutation in both probands. (XLSX 16 kb)
